# Neurosurgical intervention for giant arachnoid cyst‐induced hydrocephalus in a teenager: Delving into Pandora's box

**DOI:** 10.1002/ccr3.9280

**Published:** 2024-08-06

**Authors:** Rahul Kumar Chaudhary, Sajjad Ahmed Khan, Aakash Khatiwada, Raihana Praween, Abinash Kumar Mandal, Surya Bahadur Parajuli

**Affiliations:** ^1^ Department of Anesthesiology and Critical Care Birat Medical College Teaching Hospital Biratnagar Morang Nepal; ^2^ Birat Medical College Teaching Hospital Biratnagar Morang Nepal; ^3^ Department of Community Medicine Birat Medical College Teaching Hospital Biratnagar Morang Nepal

**Keywords:** arachnoid cyst, neurodiagnostic challenges, neurosurgical intervention, obstructive hydrocephalus, suboccipital craniotomy

## Abstract

Early recognition and prompt surgical intervention are crucial in managing giant arachnoid cysts causing obstructive hydrocephalus, as illustrated in this case of a 17‐year‐old male. Timely treatment can alleviate symptoms and prevent neurologic complications, ensuring favorable outcomes in affected patients.

## 1 INTRODUCTION

Arachnoid cysts are benign congenital lesions that arise from the arachnoid membrane of the central nervous system, containing cerebrospinal fluid (CSF). They are often asymptomatic but can present with symptoms due to mass effect or obstruction of CSF flow.^1^ These cysts typically occur in various locations, including the cerebellopontine angle (CPA), where they may lead to obstructive hydrocephalus.^2^ Hydrocephalus is a condition characterized by abnormal accumulation of CSF within the ventricular system of the brain, resulting in increased intracranial pressure (ICP).^3^ Obstructive hydrocephalus occurs when the flow of CSF is blocked, either by structural lesions such as tumors or cysts, or by anatomical abnormalities.^4^

We present a case of a 17‐year‐old male with a giant arachnoid cyst located in the prepontine region, extending to the interpeduncular and adjacent suprasellar cisterns. The patient exhibited symptoms including severe headache, vomiting, and neurologic deficits, indicative of obstructive hydrocephalus. Diagnostic imaging revealed compression of the adjacent ventricular system and midbrain, confirming the clinical suspicion.^5^ Surgical intervention, such as cyst fenestration, is often necessary to relieve the obstruction and restore normal CSF dynamics.^6^ This case report highlights the diagnostic challenges and management strategies involved in treating arachnoid cyst‐induced obstructive hydrocephalus, emphasizing the role of timely surgical intervention in improving patient outcomes.

## 2 CASE HISTORY/EXAMINATION

A 17‐year‐old male presented with a sudden onset severe headache for 7 days localized over the left temporoparietal region, continuous, non‐radiating, and associated with dizziness. He also reported frequent projectile vomiting immediately after food intake, occurring 4–5 times daily for the past 5 days. Vomitus was copious, non‐blood stained and non‐bilious. Concurrently, he noted redness and swelling of the left eye over the past 2 days. His medical history included recurrent severe headaches over the past 4–5 months, lasting approximately 80 min each episode and resolving spontaneously. Additionally, he had a history of left eye ptosis 13 years ago, with incomplete vision recovery.

On examination, the patient appeared lethargic with signs of dehydration. There were no signs of pallor, icterus, clubbing, or cyanosis. Vital signs were within normal limits. Cardiovascular and respiratory examinations were unremarkable. Abdominal examination revealed a smooth, non‐tender abdomen with normal bowel sounds. Neurologic examination showed neck rigidity with fluctuating cognition and questionable seizures without any other focal deficits.

## 3 METHODS (DIFFERENTIAL DIAGNOSIS, INVESTIGATIONS, AND TREATMENT)

Laboratory investigations revealed elevated prothrombin time (36 s), bilirubin (1.70 mg/dL), and alkaline phosphatase (213 IU/L), suggestive of possible hepatic dysfunction. Similarly, complete blood count (CBC) revealed red blood cell count of 3.7 × 10^12^/L (Reference range: 4.2 × 10^12^/L–6.5 × 10^12^/L), packed cell volume of 35.0% (Reference range: 38%–54%), mean corpuscular hemoglobin of 32.3 pg (Reference range: 25 pg–32 pg), red cell distribution width of 14.4% (Reference range: 11.5%–14%), and eosinophil count was found to be 9% (Reference range: 1%–6%). However, other blood and urine tests were within normal limits.

**FIGURE 1:**
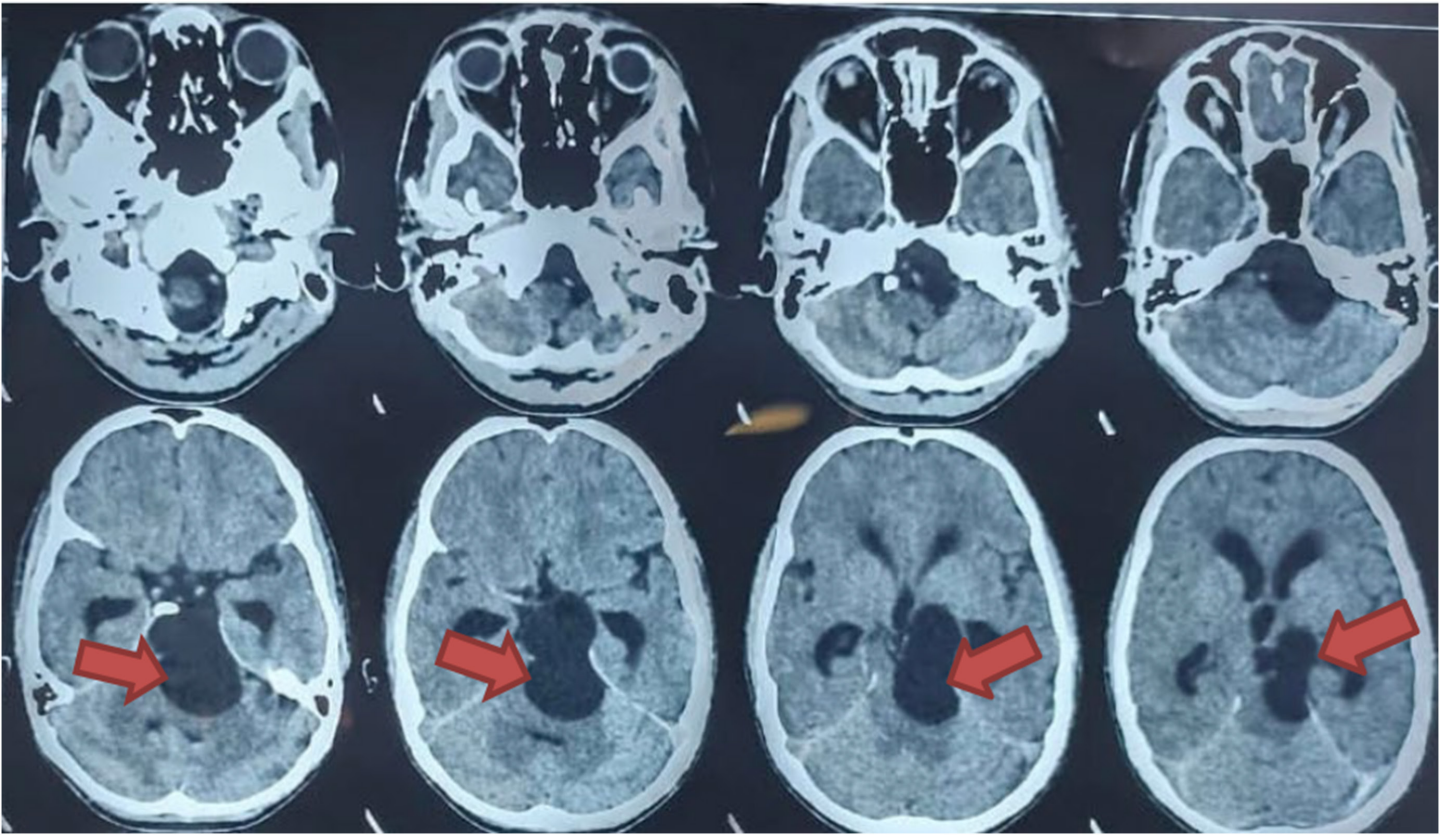
Computed tomography (CT) imaging with findings suggestive of a giant arachnoid cyst causing communicating hydrocephalus.

**FIGURE 2:**
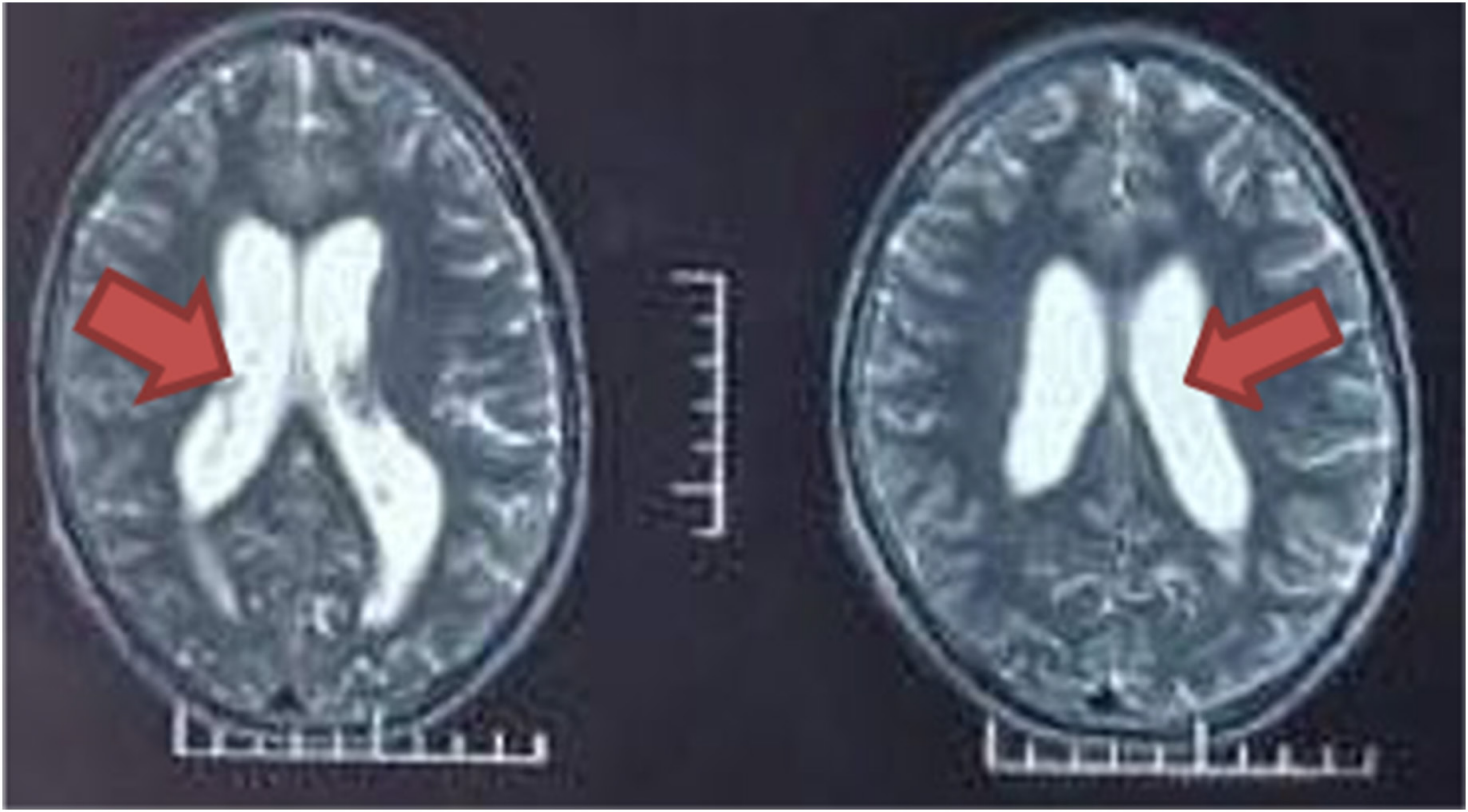
Magnetic resonance imaging (MRI) showing dilated ventricles due to hydrocephalus.

A computed tomography (CT) imaging was done and findings were suggestive of a giant arachnoid cyst causing communicating hydrocephalus (Figure 1). For confirmation of the diagnosis, contrast‐enhanced MRI of the head was performed, revealing a large, well‐defined lesion (~38 × 32 × 65 mm) in the prepontine region extending to adjacent cisterns. The lesion appeared isointense to CSF on T1, T2, and fluid‐attenuated inversion recovery (FLAIR) sequences, with no contrast enhancement or diffusion restriction (Figure 2). It caused significant compression and displacement of adjacent neurovascular structures, consistent with a giant arachnoid cyst causing communicating hydrocephalus.

After preoperative optimization of the patient surgery was planned on the third day of admission. The patient underwent suboccipital craniotomy and fenestration of the arachnoid cyst, which confirmed communication with the subarachnoid space. Postoperatively, the patient showed improvement in symptoms, and follow‐up imaging demonstrated resolution of hydrocephalus.

## 4 CONCLUSION AND RESULT (OUTCOME AND FOLLOW‐UP)

Following surgical intervention, the patient's severe headache and vomiting resolved, and the redness and swelling of the left eye subsided. There was improvement in the cognition of patient and he did not experience any seizure thereafter. Neurologic examination postoperatively showed improvement without new deficits. Repeat CT head done after 10 days as a routine follow‐up protocol confirmed adequate decompression of the cyst and resolution of hydrocephalus. The patient was discharged with appropriate follow‐up plans to monitor for recurrence or complications.

This case highlights the importance of prompt diagnosis and surgical management in patients presenting with symptoms of obstructive hydrocephalus due to arachnoid cysts, emphasizing the role of imaging and multidisciplinary approach in achieving favorable outcomes.

## 5 DISCUSSION

The presented case highlights several key clinical and diagnostic aspects relevant to arachnoid cysts in the cerebellopontine angle (CPA), particularly when complicated by obstructive hydrocephalus. Arachnoid cysts are fluid‐filled sacs lined with arachnoid membrane, located within the subarachnoid space.^7^ They are typically congenital and asymptomatic, but may become symptomatic due to mass effect, hemorrhage, or obstruction of cerebrospinal fluid (CSF) flow.^8^ In this case, the patient's symptoms of severe headache, vomiting, and neurologic deficits were indicative of increased intracranial pressure secondary to obstructive hydrocephalus caused by compression of the fourth ventricle and adjacent structures.

Diagnostic imaging, such as contrast‐enhanced MRI, remains pivotal in evaluating arachnoid cysts. The MRI findings in our patient revealed a large, well‐defined lesion predominantly in the prepontine region extending to adjacent cisterns, with characteristics typical of arachnoid cysts—isointense to CSF on T1, T2, and FLAIR sequences, and no post‐contrast enhancement.^5^ The cyst's extension into the interpeduncular and suprasellar cisterns, and compression of vital structures including the optic chiasm and cerebral peduncle, underscored the severity of mass effect.

Surgical intervention, such as suboccipital craniotomy and cyst fenestration, is the cornerstone of treatment for symptomatic arachnoid cysts causing hydrocephalus.^2^ Fenestration aims to establish communication between the cyst and subarachnoid space, allowing for normalization of CSF dynamics and alleviation of hydrocephalus. Intraoperative confirmation of the cyst's communication and subsequent improvement in symptoms and radiologic findings postoperatively validate the efficacy of this approach in our patient.

The clinical course of arachnoid cysts varies, and long‐term outcomes depend on the size, location, and extent of the cyst, as well as the promptness and success of surgical intervention.^6^ Follow‐up monitoring is crucial to detect any recurrence or complications.

Similar cases documented in medical literature underscore the diagnostic and therapeutic challenges posed by these cysts. For instance, El Damaty et al. reported on the management strategies in cases of giant arachnoid cysts presenting with hydrocephalus, emphasizing the importance of timely intervention to alleviate symptoms and prevent long‐term complications.^9^ Additionally, Nair et al. described a case where a giant arachnoid cyst led to focal neurologic deficits, highlighting the diverse clinical presentations and the need for tailored surgical approaches.^10^ The rarity of such cases is further highlighted by the distinct radiological features observed, typically showing the cysts as isointense to cerebrospinal fluid on MRI sequences without enhancement or diffusion restriction.^1^ Management strategies, including suboccipital craniotomy and cyst fenestration as performed in this case, have demonstrated efficacy in alleviating symptoms and improving patient outcomes.^9^, ^10^, ^11^ Long‐term follow‐up is crucial to monitor for recurrence and ensure sustained clinical improvement.

When arachnoid cysts are not promptly resected, they can lead to obstructive hydrocephalus, a condition where cerebrospinal fluid (CSF) flow is impeded, resulting in increased intracranial pressure. Initially, patients may experience symptoms such as severe headaches, nausea, vomiting, and visual disturbances due to this pressure buildup.^12^ Without intervention, hydrocephalus can progress, causing more severe manifestations including altered mental status, gait disturbances, and focal neurologic deficits as pressure continues to affect different brain regions.^13^ Severe untreated cases may even lead to brain herniation, a life‐threatening complication where brain tissue is displaced through openings in the skull.^14^ Managing arachnoid cysts associated with obstructive hydrocephalus presents several challenges. Diagnosing these cysts can be difficult due to varied symptomatology and potential overlap with other conditions, necessitating advanced imaging such as MRI for accurate assessment.^12^ Determining the optimal timing for surgical intervention is critical to prevent irreversible neurologic damage or complications from prolonged elevated intracranial pressure.^12^ Surgical approaches, such as endoscopic fenestration or cystoperitoneal shunting, aim to alleviate CSF accumulation and reduce pressure, but the choice of technique depends on cyst characteristics and patient‐specific factors.^11^, ^12^ Long‐term monitoring is essential post‐surgery to detect recurrence or complications, ensuring ongoing management and optimizing patient outcomes.^12^

In conclusion, this case underscores the importance of early recognition, comprehensive diagnostic evaluation with MRI, and timely surgical management in patients presenting with symptoms suggestive of obstructive hydrocephalus due to arachnoid cysts at the CPA. Such an approach is pivotal in achieving favorable outcomes and minimizing neurologic sequelae.

## 6 CONCLUSION

This case highlights challenges posed by a giant arachnoid cyst causing obstructive hydrocephalus in a 17‐year‐old male. Symptoms included acute severe headache, vomiting, and left eye issues, indicating raised intracranial pressure. Imaging revealed a large cyst in critical brain regions, prompting urgent suboccipital craniotomy and cyst fenestration, which successfully alleviated symptoms and stabilized neurologic deficits. This underscores the importance of early diagnosis, thorough evaluation, and prompt surgical intervention, emphasizing interdisciplinary collaboration for optimal outcomes.

## AUTHOR CONTRIBUTIONS

**Rahul Kumar Chaudhary:** Conceptualization; writing – review and editing. **Sajjad Ahmed Khan:** Conceptualization; writing – original draft. **Aakash Khatiwada:** Writing – original draft. **Raihana Praween:** Data curation. **Abinash Kumar Mandal:** Data curation. **Surya Bahadur Parajuli:** Writing – review and editing.

## FUNDING INFORMATION

None.

## CONFLICT OF INTEREST STATEMENT

The authors have no conflict of interest to declare.

## CONSENT

Written informed consent was obtained from the patient to publish this report in accordance with the journal's patient consent policy.

## Data Availability

Data will be provided by the corresponding author upon reasonable request. Images uploaded in the separate files.
